# Adding Acotiamide to Gastric Acid Inhibitors Is Effective for Treating Refractory Symptoms in Patients with Non-erosive Reflux Disease

**DOI:** 10.1007/s10620-018-5377-9

**Published:** 2018-11-21

**Authors:** Hiroshi Yamashita, Akihiko Okada, Kohji Naora, Masafumi Hongoh, Yoshikazu Kinoshita

**Affiliations:** 1 Department of Gastroenterology and Hepatology, Saiseikai Nakatsu Hospital, 2-10-39 Shibata Kita-ku, Osaka, 530-0012 Japan; 2 Department of Pharmacy, Shimane University Hospital, 89-1 Enyacho, Izumo, Shimane 693-0021 Japan; 3 Department of Gastroenterology and Hepatology, Shimane University Hospital, 89-1 Enyacho, Izumo, Shimane 693-0021 Japan

**Keywords:** Acotiamide, Overall treatment effect, Regurgitation, Multiple intraluminal impedance-pH monitoring, Proximal reflux, Acid reflux

## Abstract

**Background:**

Approximately 30% of patients who are treated with proton pump inhibitors (PPIs) for gastroesophageal reflux disease (GERD) experience persistent symptoms. No prokinetic agent regiments are useful for symptom relief.

**Aims:**

This study was conducted to examine the effect of adding acotiamide to PPI or vonoprazan refractory GERD.

**Methods:**

This was a randomized, prospective, double-blind, placebo-controlled trial. Seventy-one patients were enrolled. Patients underwent upper endoscopy before initial therapy [15 reflux esophagitis and 55 non-erosive reflux disease (NERD)]. Patients with persistent reflux symptoms were administered 300 mg/day acotiamide or placebo for 2 weeks. The primary endpoint was overall treatment effect (OTE), and gastrointestinal symptoms were evaluated. High-resolution manometry (HRM) and 24-h multiple intraluminal impedance-pH (MII-pH) monitoring were conducted before and after treatment when possible.

**Results:**

Seventy patients were randomized (35 acotiamide and 35 placebo). Sixteen and 10 patients in the acotiamide and placebo groups, respectively, completed MII-pH and HRM. The OTE improvement rates were 28.6% and 14.3% in patients administered acotiamide and placebo, respectively (*p *= 0.145). In patients with NERD, however, the OTE improvement rate and responder rate for regurgitation in the acotiamide group was significantly higher than those in the placebo group (29.6 vs. 7.1%; *p* = 0.030, 37.0 vs. 10.7%; *p *= 0.021, respectively). Acotiamide significantly reduced the total reflux episodes (*p* = 0.001), acid (*p *= 0.020), proximal reflux (*p* = 0.007), and liquid reflux (*p* = 0.013) episodes.

**Conclusion:**

Adding acotiamide to gastric acid inhibitors can improve symptoms in patients with refractory NERD.

## Introduction

Proton pump inhibitors (PPIs) are widely used to treat patients with gastroesophageal reflux disease (GERD). However, 20–30% of patients treated with PPIs for GERD experience persistent heartburn and/or regurgitation [1, 2], resulting in a lower quality of life of patients. Because GERD is one of the most prevalent chronic diseases worldwide, managing these refractory patients is a substantial challenge for general clinicians and gastroenterologists.

Recent studies using multichannel intraluminal impedance-pH (MII-pH) monitoring have shown that refractory symptoms are often associated with weakly acidic reflux events and/or proximal reflux [3, 4]. It is well known that most reflux episodes occur during transient lower esophageal sphincter relaxation (TLESR) [5]. Moreover, TLESRs are affected by slow gastric emptying and impaired gastric accommodation [6, 7]. Ishii et al. [8] investigated the relationship between reflux characteristics and delayed gastric emptying and found that severe delayed gastric emptying was related to increased non-acid reflux leading up to the proximal esophagus, suggesting that gastrointestinal motility plays an important role in generating reflux and GERD symptoms. Thus, prokinetic drugs may improve reflux symptoms by augmenting gastric motility via reducing the number of TLESRs and reflux events.

Notably, some regimens using prokinetic agents such as mosapride [9], prucalopride [10], and revexepride [11] have been examined for their ability to alleviate PPI-refractory symptoms, but no regimens were shown to be useful for symptom relief. Acotiamide, a new prokinetic agent, improves slow gastric emptying and impaired gastric accommodation [12–14]. We also reported that acotiamide reduced the number of TLESRs and TLESRs related to reflux in healthy subjects [15], suggesting its beneficial effects on GERD. However, few studies have examined the effect of acotiamide in patients with PPI-refractory GERD. Therefore, we assessed the effect of acotiamide on symptomatic improvement, esophageal reflux parameters, and esophageal contraction characteristics in patients with gastric acid inhibitor-refractory GERD.

## Methods

### Study Design

This was a randomized, prospective, double-blind, placebo-controlled trial conducted from September 2015 to March 2018 in the Department of Gastroenterology, Saiseikai Nakatsu Hospital. This study was performed in accordance with the clinical principles of the Declaration of Helsinki. The study protocol was approved by the Institution Review Board of Saiseikai Nakatsu Hospital and was registered with the University Hospital Medical Information Network (UMIN) Clinical Trial Registry as UMIN No. 000026364. Informed consent was obtained from all patients.

The objectives of this study were to determine the effects of: (1) adding acotiamide to gastric acid inhibitors (vonoprazan or PPIs) on the overall treatment effect (OTE) rate and reflux symptoms (heartburn or regurgitation) improvement rate; (2) acotiamide on esophageal manometric parameters derived from high-resolution manometry (HRM); and (3) acotiamide on esophageal reflux parameters derived from 24-h MII-pH.

### Patients

Seventy-one patients who complained of typical GERD symptoms (heartburn and/or regurgitation) of at least moderate severity at a mean frequency of more than twice per week despite treatment with a standard dose of PPIs or vonoprazan for at least 8 weeks were enrolled. All patients underwent upper endoscopy before starting initial PPI therapy, which revealed that 15 patients had reflux esophagitis (RE) (5 grade A, 6 grade B, 2 grade C, and 2 grade D, according to LA classification), while the remaining patients had non-erosive reflux disease (NERD).

Patient profiles (age, gender, body mass index, pretreatment regimen, alcohol intake, and smoking) and *Helicobacter pylori* infection status were compared. Patients with peptic ulcers or gastric or esophageal malignancy or those who underwent successful eradication of *H. pylori* within the previous 6 months or gastrectomy were excluded. Additionally, patients who were currently being treated with another prokinetic agent, non-steroidal anti-inflammatory drugs, and low-dose aspirin were excluded.

### Study Protocol

Before randomization, all patients were invited to undergo further HRM and 24-h MII-pH at baseline and after 2 weeks of treatment if possible. Randomization was performed using a computer-generated program (RANDBETWEEN software, Microsoft, Redmond, WA, USA). Eligible patients were assigned a randomization number according to a predetermined list. These numbers were allocated in sequential order and registered in the patient enrollment list and allocation was concealed to both investigators and patients. Patients were randomized to receive either 100 mg acotiamide or placebo to be administered three times daily 30 min before each meal for 2 weeks.

Acotiamide (100 mg) and placebo were capsuled to ensure that they were visually indistinguishable and were provided in identical medication boxes. Additionally, patients continued their stable gastric acid suppressive treatment regimen (maintained at the same dose and type of drugs during the study). Symptoms and impedance-pH/manometry data were assessed at baseline and after 2 weeks of treatment when possible. The investigator was blinded to the type of study drugs for investigators to prevent bias. All patients reported adverse events during the study period.

### Symptom Assessments

We used global assessment of OTE questionnaires completed by the participants at 1 and 2 weeks after treatment for the primary endpoint as recommended by the Rome guidelines [16]. The following question was asked: ‘How were your reflux symptoms during the last week in comparison with the baseline period?’ Answers were scored on a 7-point Likert scale as follows: 1, extremely improved; 2, improved; 3, slightly improved; 4, unchanged; 5, slightly aggravated; 6, aggravated; and 7, extremely aggravated. Grades 1 or 2 indicated that the therapy was effective. We assessed the effective OTE rate at 2 weeks after treatment.

We also evaluated each gastrointestinal symptom using questionnaires that included 11 items; heartburn, regurgitation, epigastric pain, epigastric burning, epigastric discomfort, abdominal fullness, early satiety, bloating, nausea, belching, and dysphagia. Severity was rated on a 4-point Likert scale from 0 to 3 (0, none; 1, mild; 2, moderate; 3, severe). The frequency was rated on a 5-point Likert scale from 0 to 4; none, once per week, 2–3 times per week, 4–6 times per week, or daily. We calculated the sum of the severity and frequency scores in each symptom and a responder was defined as a patient showing a greater than 50% decrease in the symptom score at 2 weeks of treatment compared to the baseline score.

### HRM and MII-pH

The Starlet HRM system (Starlet, Star Medical, Inc., Tokyo, Japan) was used. This system is equipped with a catheter and 36 solid-state sensors spaced at 1-cm intervals (Unisensor AG, Attikon, Switzerland). The manometric protocol included 10 swallows of 5 mL of water at 30-s intervals in the supine position. Manometric data were analyzed using software from Star Medical, Inc. We evaluated lower esophageal sphincter pressure, integrated relaxation pressure, distal esophageal contractile integral, contractile front velocity, and percent successful peristaltic rate according to Chicago criteria [17].

The data derived from impedance-pH monitoring (Sleuth; Sandhill Scientific, Highlands Ranch, CO, USA) were analyzed manually using dedicated software (Bioview Analysis; Sandhill Scientific). Episodes of liquid-only reflux were identified by a retrograde decrease in impedance from baseline by 50% at a minimum of two sites. Episodes of mixed liquid–gas reflux were defined as gas reflux occurring immediately before or during liquid reflux. Reflux episodes detected by impedance were classified as acid reflux episodes if the pH sensor recorded a decrease in pH to < 4 for more than 5 s and were classified as weakly acidic reflux episodes if the pH remained > 4. The percentage time of esophageal pH < 4 (pH holding time ratio) was calculated in a similar manner. The bolus clearance time was defined as the duration between the time when the impedance value decreased to < 50% of the baseline value and the time required to reach 50% of the baseline value. A symptom was considered as associated with reflux if a reflux episode was detected within 5 min before symptom occurrence.

### Statistical Analysis

There are no previous data regarding the use of acotiamide for treating refractory GERD. The sample size of our study was calculated by assuming that the improvement rate would be 10% in the placebo group and 30% in the acotiamide group according to a previous phase III trial of acotiamide [18]. Thus, 50 patients, including a 10% drop out rate, were required in each group to detect a significant difference at a 5% significance level and statistical power of 80%.

Efficacy analysis was based on the full analysis set population. The proportion of responders using OTE as the primary efficacy variable and improvement rate of each symptom were evaluated using the *χ*^2^ test. The manometric and MII-pH values were compared between baseline and post-treatment using the Wilcoxon rank-sum test. We employed the *t* test or by the *χ*^2^ to compare background factors. Data were expressed as the median (interquartile range), and values of *p *< 0.05 were considered significant.

## Results

### Enrollment and Baseline Characteristics of the Patients

**Fig. 1:**
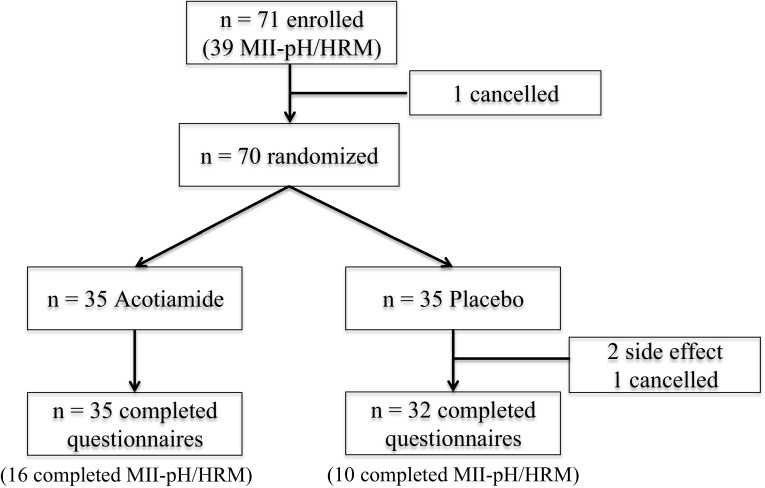
Flow diagram of enrolled subjects. Seventy-one patients were enrolled. One patient withdrew from the study before randomization and 70 eligible patients were randomized to the acotiamide group (*n* = 35) and placebo group (*n* = 35). Finally, 35 patients in the acotiamide group and 32 patients in the placebo group completed the study. Sixteen patients in the acotiamide group and 10 patients in the placebo group had MII-pH/HRM at baseline and after 2 weeks

A flow diagram showing the process of subject enrollment is shown in Fig. 1. One patient withdrew from the study before randomization and a total of 70 patients were randomized (35 each in the acotiamide and placebo groups) before analysis. There was no difference in the types of pretreatment gastric acid inhibitors between the two groups (all patients with RE were administered vonoprazan and all patients with NERD were administered any PPIs). All patients were administered at least one dose of the investigational product and were therefore included in the safety population. Of the 70 patients in the safety population, 67 patients (35 acotiamide, 32 placebo) completed the study. Twenty patients in the acotiamide group and 19 patients in the placebo group agreed to undergo MII-pH and HRM at baseline. Sixteen of the 20 patients in the acotiamide group and 10 of the 19 patients in the placebo group agreed to MII-pH and HRM after 2 weeks of treatment. There were no significant differences in the baseline characteristics between groups (Table 1).

**Table 1:** Patient characteristics

	Acotiamide (*n* = 35)	Placebo (*n* = 35)	*p* value
Age, years (median)	70 (38–83)	63 (22–86)	0.078
Gender (Female, %)	19 (54.2%)	18 (51.4%)	0.810
BMI, kg/cm^3^ (median)	22.0 (16.5–28.4)	21.1 (16.3–34.0)	0.959
Pretreatment gastric inhibitors			
Vonoprazan	8	7	
PPIs (LPZ/RPZ/EPZ)	27 (7/10/10)	28 (8/12/8)	0.770
LA classification (M/A/B/C/D)	27/1/4/2/1	28/4/2/0/1	0.151
*Helicobacter pylori* infection (Yes/No)	11/24	8/27	0.420
Smoking (Yes/No)	3/32	5/30	0.452
Alcohol (Yes/No)	7/28	13/22	0.112

*LPZ* lansoprazole, *RPZ* rabeprazole, *EPZ* esomeprazole

### Symptomatic Efficacy

**Fig. 2:**
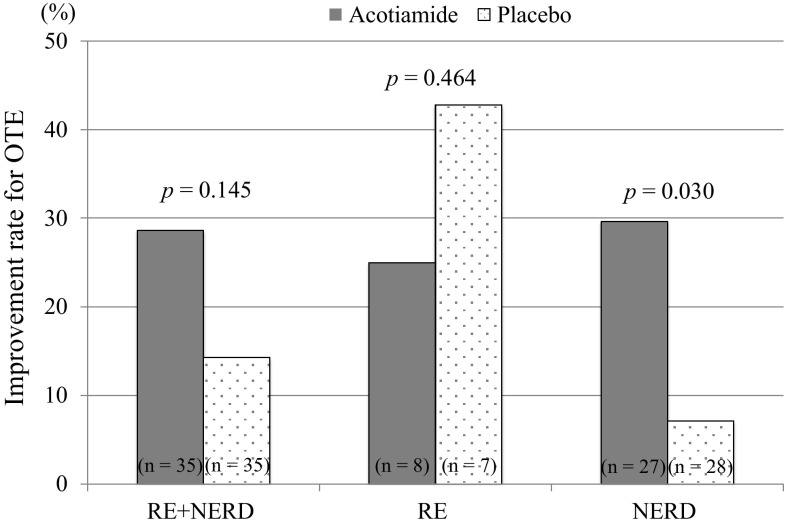
The responder rate based on the OTE at 2 weeks was 28.6% in patients administered acotiamide and 14.3% in those administered the placebo (*p *= 0.145). Patients with RE showed no significant difference in the OTE improvement rate (25.0 vs. 42.8%*, p* = 0.464), while patients with NERD showed a significantly higher improvement rate than the placebo group (29.6 vs. 7.1%, *p* = 0.030)

The responder rate based on the OTE at 2 weeks was 28.6% for patients administered acotiamide and 14.3% for patients administered placebo. No significant difference was found between the acotiamide and placebo groups (*p *= 0.145). Furthermore, we conducted subgroup analysis of patients with RE and NERD. In patients with RE (8 acotiamide and 7 placebo), there was no significant difference between the acotiamide group and placebo group (25.0 vs. 42.8%*, p* = 0.464). In patients with NERD (27 acotiamide and 28 placebo), the OTE improvement rate in the acotiamide group was significantly higher than that in the placebo group (29.6 vs. 7.1%, *p* = 0.030) (Fig. 2).

**Fig. 3:**
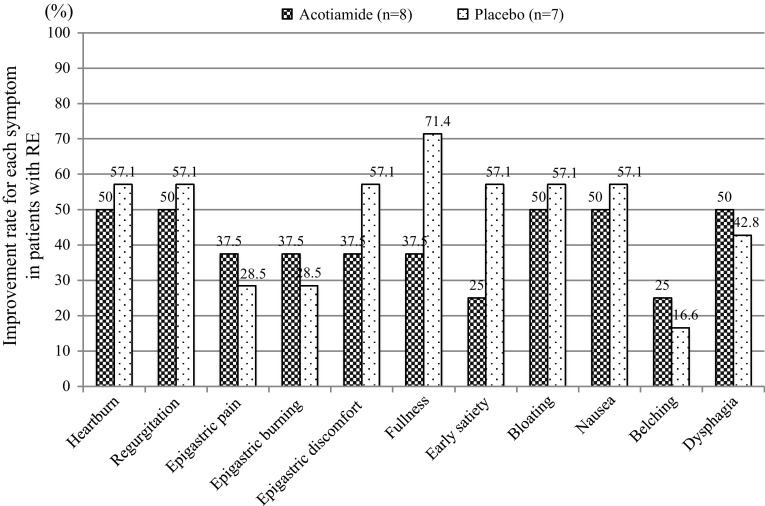
In patients with RE, there were no significant differences in each symptom between the acotiamide and placebo groups

**Fig. 4:**
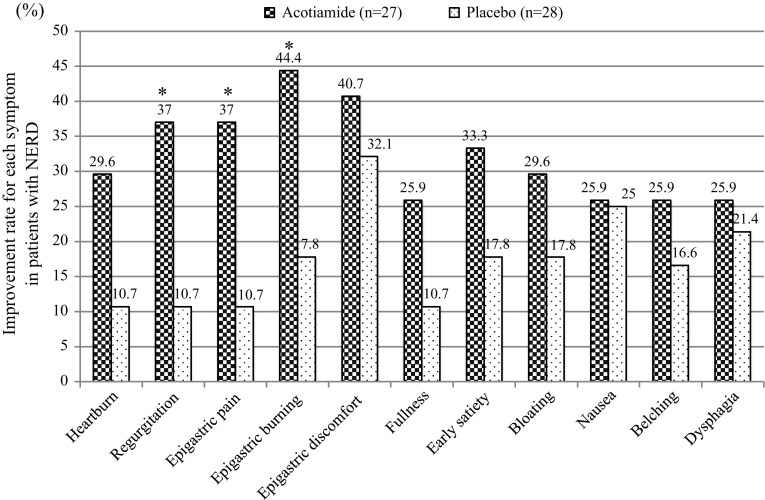
In patients with NERD, the responder rates for regurgitation, epigastric pain, and epigastric burning were significantly higher in the acotiamide group than in the placebo group (37.0 vs. 10.7%; *p *= 0.021, 37.0 vs. 10.7%; *p *= 0.032, and 44.4 vs. 17.8%; *p *= 0.021, respectively). *p < 0.05

For the responder rates of all symptoms according to RE and NERD, in patients with RE, there were no significant differences between the acotiamide and placebo groups for each symptom (heartburn (50.0 vs. 57.1%; *p *= 0.782), regurgitation (50.0 vs. 57.1%; *p* = 0.782), epigastric pain (37.5 vs. 28.5%; p = 0.714), epigastric burning (37.5 vs. 28.5%; *p* = 0.714), epigastric discomfort (37.5 vs. 57.1%; *p *= 0.446), abdominal fullness (37.5 vs. 71.4%; *p *= 0.188), early satiation (25.0 vs. 57.1%; *p *= 0.204), abdominal bloating (50.0 vs. 57.1%; *p *= 0.782), nausea (50.0 vs. 57.1%; *p *= 0.782), belching (25.0 vs. 16.6%; *p *= 0.604), and dysphagia (50.0 vs. 42.8%; *p *= 0.782) (Fig. 3). In contrast, in patients with NERD patients, the responder rates for regurgitation, epigastric pain, and epigastric burning were significantly higher in the acotiamide group than in the placebo group (37.0 vs. 10.7%; *p *= 0.021, 37.0 vs. 10.7%; *p *= 0.032, and 44.4 vs. 17.8%; *p *= 0.021, respectively). Acotiamide showed no significant difference compared to the placebo in the individual response rate for heartburn (29.6 vs. 10.7%; *p *= 0.079), epigastric discomfort (40.7 vs. 32.1%; *p *= 0.507), abdominal fullness (25.9 vs. 10.7%; *p *= 0.143), early satiation (33.3 vs. 17.8%; *p *= 0.187), abdominal bloating (29.6 vs. 17.8%; *p *= 0.304), nausea (25.9 vs. 25.0%; *p *= 0.934), belching (25.9 vs. 16.6%; *p *= 0.280), and dysphagia (25.9 vs. 21.4%; *p *= 0.750) (Fig. 4).

### Stationary HRM

Stationary HRM at both baseline and 2 weeks of treatment was performed for 16 patients administered acotiamide and 10 patients administered placebo (Table 2). Lower esophageal sphincter pressure as measured during the respiratory minimum was not affected by acotiamide, while integrated relaxation pressure treated with acotiamide was significantly higher than at baseline [8.9 (0–27) vs. 10.9 (0–29.6), *p* = 0.023]. Acotiamide did not affect distal esophageal contractile integral, contractile front velocity, and percentage of successful peristalsis. Peristaltic break treated with acotiamide was significantly shorter than at baseline [3.1 (0–16.1) vs. 0.8 (0–17), *p* = 0.020]. No significant differences were found in the placebo group for any parameters between baseline and 2 weeks.

**Table 2:** Changes in manometric characteristics (acotiamide; *n* = 16, placebo; *n* = 10)

		Baseline	Post-treatment	*p* value
LES pressure (median; mmHg)	Acotiamide	10.7 (0.7–46.7)	16.0 (0–48.4)	0.410
	Placebo	16.1 (0.6–38.2)	17.8 (3.8–27)	0.332
IRP4s (mmHg)	Acotiamide	8.9 (0–27.4)	10.9 (0–29.6)	0.023
	Placebo	11.5 (3.5–26.8)	12.9 (3.7–17)	0.414
Peristaltic break (cm)	Acotiamide	3.1 (0–16.1)	0.8 (0–17)	0.020
	Placebo	0.1 (0–4.1)	0.2 (0–14.1)	0.362
DCI (mmHg cm s)	Acotiamide	1500.1 (349.1–8935.9)	3036.1 (238.2–9174.1)	0.410
	Placebo	3858.1 (961.7–8158.7)	3693.5 (953.4–10,118.1)	0.759
CFV (cm/s)	Acotiamide	3.2 (2.5–26.9)	4.1 (0–8.39	0.162
	Placebo	3.1 (2.3–5.1)	3.5 (1.8–6.1)	0.358
% Success peristalsis	Acotiamide	100 (0–100)	100 (0–100)	0.199
	Placebo	100 (40–100)	100 (20–100)	0.845

Data were expressed as median (interquartile range)

*LES* lower esophageal sphincter, *IRP* integrated relaxation pressure, *DCI* distal contractile integral, *CFV* contractile front velocity

### 24-h Impedance-pH Monitoring

Twenty-four-hour impedance-pH monitoring at both baseline and 2 weeks of treatment was conducted for 16 patients administered acotiamide and 10 patients administered placebo (Table 3). Acotiamide significantly reduced total reflux episodes [39.5 (6–79) vs. 29.0 (7–52), *p* = 0.001]. Impedance-pH monitoring revealed that acotiamide significantly reduced acid reflux [13.5 (1–42) vs. 3.5 (0–20), *p *= 0.020] and liquid reflux [19.0 (0–40) vs. 12.0 (1–24), *p* = 0.013] episodes; however, the differences in weakly acidic reflux [20.5 (1–42) vs. 16 (4–49), *p* = 0.064] and mixed reflux [20.0 (3–58) vs. 14.0 (4–43), *p* = 0.057] episodes were not significant. Proximal reflux episodes and distal reflux episodes were significantly reduced [17.5 (2–46) vs. 13.0 (3–33); *p* = 0.007, 21.0 (4–41) vs. 14.5 (4–30); *p *= 0.047, respectively]. Furthermore, the bolus clearance time, as measured by impedance monitoring, was not affected by acotiamide [12.5 (3–58) vs. 14.0 (4–43), *p* = 0.816]. In contrast, no significant differences were found in the placebo group for any parameters between baseline and 2 weeks.

**Table 3:** Changes in reflux characteristics (acotiamide; *n* = 16, placebo; *n* = 10)

		Baseline	Post-treatment	*p* value
Esophageal % pH < 4 holding time	Acotiamide	0.35 (0–17.2)	0.20 (0–65.9)	0.711
	Placebo	0.20 (0–5.2)	0.10 (0–4.6)	0.271
Total reflux episodes	Acotiamide	39.5 (6–79)	29.0 (7–52)	0.001
	Placebo	46.0 (18–112)	34.0 (15–95)	0.153
Acid reflux episodes	Acotiamide	13.5 (1–42)	3.5 (0–20)	0.020
	Placebo	4.0 (0–19)	2.0 (0–42)	0.787
Weakly acidic reflux episodes	Acotiamide	20.5 (4–62)	16.0 (4–49)	0.064
	Placebo	44.0 (17–109)	31.0 (4–93)	0.540
Proximal reflux episodes	Acotiamide	17.5 (2–46)	13.0 (3–33)	0.007
	Placebo	20.0 (13–64)	19.5 (6–44)	0.259
Distal reflux episodes	Acotiamide	21.0 (4–41)	14.5 (4–30)	0.047
	Placebo	20.0 (13–64)	24.0 (5–62)	0.192
Liquid reflux episodes	Acotiamide	19.0 (0–40)	12 (1–24)	0.013
	Placebo	13.5 (8–61)	15.0 (3–61)	0.400
Mixed reflux episodes	Acotiamide	20.0 (3–58)	14.0 (4–43)	0.057
	Placebo	21.5 (8–68)	23.0 (12–44)	0.138
Bolus clearance time (s)	Acotiamide	12.5 (4–38)	14.5 (6–54)	0.816
	Placebo	11.5 (6–100)	15.5 (6–29)	0.233
Symptom associated reflux episodes	Acotiamide	1.0 (0–45)	0 (0–8)	0.035
	Placebo	2.5 (80–59)	3.5 (0–35)	0.058

Data were expressed as median (interquartile range)

### Adverse Events

Two patients in the placebo group discontinued the study because of treatment-emergent adverse events (nausea) and one patient discontinued because of worsened epigastric discomfort compared to that at baseline. No treatment-emergent adverse effects were observed in the acotiamide group.

## Discussion

This is the first study to assess the efficacy of adding acotiamide to gastric acid inhibitors to treat patients with refractory GERD in a placebo-controlled double-blind manner. An appropriate therapeutic approach has not been established for patients with GERD who failed to respond to acid suppression therapy. Increasing the dosage of an ordinary PPI from once to twice daily is a common strategy in clinical practice for patients showing a partial response to PPIs. However, the efficacy of dosage escalation is limited, particularly for patients with NERD [19]. Therefore, it is important to determine the efficacy of therapeutic agents added to PPI therapy. A drug with a prokinetic effect on the upper gastrointestinal tract is thought to exert its benefits by affecting gastric or esophageal motility, but few drugs show these effects.

Acotiamide inhibits the activity of acetylcholinesterase and enhances acetylcholine release by antagonizing the muscarinic M1 and M2 receptors, which improves delayed gastric emptying and impaired gastric accommodation, and acotiamide showed a significant response to functional dyspepsia symptoms [14, 18, 20]. Here, acotiamide did not significantly change OTE improvement as a primary endpoint, while in subgroup analysis acotiamide showed a significant effect compared to the placebo in patients with NERD. Furthermore, analysis of the improvement rate for each symptom showed that acotiamide significantly improved regurgitation, epigastric pain, and epigastric burning scores in patients with NERD.

GERD patients often have dyspeptic symptoms such as epigastric pain and postprandial fullness, and particularly epigastric pain or burning symptom have been found to be a significant factor in insufficient therapeutic efficacy [21]. The rate of dyspeptic symptoms that were more than moderate in our study were 40.0% and 56.3% in patients with RE and NERD, respectively (data not shown), suggesting that patients with overlapping GERD and FD were enrolled. A previous study was conducted to evaluate the effect of acotiamide in patients with FD with postprandial distress syndrome (PDS) type symptoms for 4 weeks. The study showed that acotiamide significantly improved postprandial fullness, upper abdominal bloating, and early satiation [18]; however, acotiamide did not significantly improve PDS symptoms in our study. This may be because of differences in the study design such as patient selection (FD-PDS vs. GERD overlapping FD) or study duration (4 vs. 2 weeks). Therefore, the significant improvement in not only regurgitation but also epigastric pain and epigastric burning by adding acotiamide may contribute to significantly improving the OTE in patients with NERD.

A significant difference in OTE improvement was observed only in patients with NERD but not in those with RE. It is well known that the severity of symptoms and low quality of life of patients with NERD are similar to those of patients with RE [22]; however, the esophageal acid exposure time of NERD is lower than that of RE [23]. These findings suggest that patients with NERD are more sensitive to acid reflux than patients with RE. In Japan, vonoprazan, which suppresses gastric acid more potently than conventional PPIs, is available for RE treatment. Vonoprazan was used to treat all patients with RE in our study, and MII-pH was conducted before adding acotiamide to determine the mean number of acid reflux events. These events were more frequent in patients with NERD than in those with RE (14.1 vs. 5.8, data not shown). Therefore, adding acotiamide to reduce gastric acid reflux may contribute to a better response in patients with NERD.

Additionally, it has been shown that regurgitation can be caused by not only acid reflux but also weak acidic reflux extended to the proximal esophagus [24–26]. Thus, there are three explanations for acotiamide’s significant improvement effects on regurgitation.

First, the reduction in proximal reflux episodes caused by administration of acotiamide may be an important factor in improving symptoms. Esophageal hypersensitivity at the proximal esophagus for refluxate contributes to persistent symptoms [27]. Moreover, Emerenziani et al. [28] evaluated reflux migration according to the acidity of the refluxate and showed that acid reflux was likely to extend higher than weakly acidic reflux. Our results showed that acotiamide significantly reduced acid reflux events; therefore, it is likely that the observed effect of acotiamide in reducing proximal reflux by significantly reducing acid reflux contributes to improving regurgitation.

Second, regarding the effect of acotiamide on esophageal motility, the peristaltic break was shortened by administration of acotiamide, which agrees with the results of a previous study showing that acotiamide reduced weak peristalsis with a small break (2–5 cm) [29]. Therefore, the effect of acotiamide on shortening the peristaltic break at the proximal esophagus may prevent reflux from extending to the more proximal esophagus.

Third, heartburn is thought to occur when acid and/or other components of the refluxed gastric content reach sensory nerve endings through mucosal dilated intercellular spaces (DIS) [30]. Farré et al. [31] investigated the DIS at the proximal esophagus by injecting several pH solutions into the distal esophagus. They demonstrated that not only acid reflux but also weakly acidic solutions exposed to the distal esophagus-induced DIS in the most proximal ‘non-exposed’ esophageal area. These data suggest that frequent distal reflux episodes irrespective of acidity generate symptoms by inducing esophageal hypersensitivity. Considering these findings, the reduction of reflux events may affect the patients’ perception of refluxate.

There were several limitations to this study. First, the relatively small number of patients here is a crucial limiting factor. This study was terminated without having reached the initial target of 50 patients per treatment arm because of the difficulty of recruiting patients at a single center. Second, the short treatment duration of 2 weeks may have affected the results. In a phase III study of acotiamide in patients with FD-PDS, a significant difference in the OTE improvement rate between the acotiamide and placebo groups was apparent from week 2. Thus, we considered that a study duration of 2 weeks was appropriate. Third, the number of patients who had MII-pH/HRM before administration of the study drugs was small, and thus those with functional heartburn whose symptoms were not associated with reflux events may have been included in the group of patients with NERD. Approximately 20% of patients with reflux symptoms have functional heartburn, and this proportion is likely higher among those who show a partial response to PPI therapy [32]. Finally, the true mechanism by which the pharmacodynamics of reflux was altered is unclear because we did not examine gastric motility. A previous study showed acotiamide enhances duodenum, small intestine, and colon contractions [33], suggesting that acotiamide plays an important role in reducing intragastric pressure by reducing bowel transit time, through which acotiamide may reduce the reflux events or volume related to refluxate migration. Additional studies of larger sample sizes and for longer treatment durations are needed to determine which factor is related to the change in reflux characteristics.

In conclusion, this placebo-controlled study of patients with refractory GERD revealed that adding acotiamide significantly improved OTE and regurgitation compared to the placebo in patients with NERD by significantly reducing total reflux episodes, particularly acid reflux or proximal reflux episodes. Crucial side effects were not reported in the acotiamide group. Thus, adding acotiamide in a complementary manner with gastric acid inhibitors may be beneficial for regurgitation-predominant patients. However, the sample size was small, and thus, a larger sample size and longer treatment duration should be examined in a multi-center study.

## References

[1] Katz PO, Zavala S (2010). Proton pump inhibitors in the management of GERD. J Gastrointest Surg.

[2] Fass R, Sifrim D (2009). Management of heartburn not responding to proton pump inhibitors. Gut.

[3] Mainie I, Tutuian R, Shay S (2006). Acid and non-acid reflux in patients with persistent symptoms despite acid suppressive therapy: a multicenter study using combined ambulatory impedance-pH monitoring. Gut.

[4] Zerbib F, Romans S, Ropert A (2006). Esophageal pH-impedance monitoring and symptom analysis in GERD: a study in patients off and on therapy. Am J Gastroenterol.

[5] Kessing BF, Conchillo JM, Bredenoord AJ (2011). Review article: the clinical relevance of transient lower oesophageal sphincter relaxations in gastrooesophageal reflux disease. Aliment Pharmacol Ther.

[6] Piche T, Galmiche JP (2005). Pharmacological targets in gastro-oesophageal reflux disease. Basic Clin Pharmacol Toxicol.

[7] Emerenziani S, Sifrim D (2005). Gastroesophageal reflux and gastric emptying, revisited. Curr Gastroenterol Rep.

[8] Ishii S, Fukahori S, Asagiri K (2017). Severe delayed gastric emptying induces non-acid reflux up to proximal esophagus in neurologically impaired patients. J Neurogastroenterol Motil.

[9] Miwa H, Inoue K, Ashida K (2011). Randomised clinical trial: efficacy of the addition of a prokinetic, mosapride citrate, to omeprazole in the treatment of patients with non-erosive reflux disease—a double-blind, placebo-controlled study. Aliment Pharmacol Ther.

[10] Kessing BF, Smout AJPM, Bennink RJ (2014). Prucalopride decreases esophageal acid exposure and accelerate gastric emptying in healthy subjects. Neurogastroenterol Motil.

[11] Tack J, Zerbib F, Blondeau K (2015). Randomized clinical trial: effect of the 5-TH4 receptor against revexepride on reflux parameters in patients with persistent reflux symptoms despite PPI treatment. Neurogastroenterol Motil.

[12] Ogishima M, Kaibara M, Ueki S (2000). Z-338 facilitates acetylcholine release from enteric neurons due to blockade of muscarinic autoreceptors in guinea pig stomach. J Pharmacol Exp Ther.

[13] Nakajima T, Nawata H, Ito Y (2000). Z-338, a newly synthetized carboxyamide derivative, stimulates gastric motility through enhancing the excitatory neurotransmission. J Smooth Muscle Res.

[14] Kusunoki H, Haruma K, Manabe N (2012). Therapeutic efficacy of acotiamide in patients with functional dyspepsia based on enhanced postprandial gastric accommodation and emptying: randomized controlled study evaluation by real-time ultrasonography. Neurogastroenterol Motil.

[15] Yamashita H, Kanamori A, Fukuchi T (2015). Novel prokinetic acotiamide reduces transient lower esophageal sphincter relaxation in healthy subjects. Digestion.

[16] Camilleri M, Drossman A, Fehnel S (2007). Primary endpoints for FGID trials: a review of binary and integrated symptoms assessments. Clin Gastroenterol Hepatol.

[17] Kahrilas PJ, Bredenoord AJ, Fox M (2015). The Chicago classification of esophageal motility disorders, v3.0. Neurogastroenterol Motil.

[18] Matsueda K, Hongo M, Tack J (2012). A placebo-controlled trial of acotiamide for meal-related symptoms of functional dyspepsia. Gut.

[19] Furuta T, Shimatani T, Sugimoto M (2011). Investigation of pretreatment prediction of proton pump inhibitor (PPI)-resistant patients with gastroesophageal reflux disease and the dose escalation challenge of PPIs-TORNADO study: a multicenter prospective study by the Acid-Related Symptom Research Group in Japan. J Gastroenterol.

[20] Nakamura K, Tomita T, Oshima T (2017). A double-blind placebo controlled study of acotiamide hydrochloride for efficacy on gastrointestinal motility of patients with functional dyspepsia. J Gastroenterol.

[21] Matsuhashi N, Kudo M, Yoshida N (2015). Factors affecting response to proton pump inhibitor therapy in patients with gastroesophageal reflux disease: a multicenter prospective observational study. J Gastroenterol.

[22] Garros A, Mion F, Marjoux S (2016). Factors associated with nonresponse to proton pump inhibitors therapy in patients referred for esophageal pH-impedance monitoring. Dis Esophagus.

[23] Bredenoord AJ, Hemmink GJM, Smout AJPM (2009). Relationship between gastro-oesophageal reflux pattern and severity of mucosal damage. Neurogastroenterol Motil.

[24] Zerbib F, Duriez A, Roman S (2008). Determinants of gastro-oesophageal reflux perception in patients with persistent symptoms despite proton pump inhibitors. Gut.

[25] Tutuian R, Mainie I, Allan R (2006). Effects of a 5-HT(4) receptor agonist on oesophageal function and gastro-oesophageal reflux: studies using combined impedance-manometry and combined impedance-pH. Aliment Pharmacol Ther.

[26] Sifrim D, Mittal R, Fass R (2007). Review article: acidity and volume of the refluxate in the genesis of gastro-oesophageal reflux disease symptoms. Aliment Pharmacol Ther.

[27] Tack J (2008). Is there a unifying role for visceral hypersensitivity and irritable bowel syndrome in non-erosive reflux disease?. Digestion.

[28] Emerenziani S, Cicala M, Zhang X (2007). Effect of oesophagitis on proximal extent of gastro-oesophageal reflux. Neurogastroenterol Motil.

[29] Hoshino S, Takenouchi N, Hanada Y (2017). Effect of acotiamide on esophageal motility in healthy subjects: a randomized, double-blind, placebo-controlled crossover study. Esophagus.

[30] Barlow WJ, Orlando RC (2005). The pathogenesis of heartburn in nonerosive reflux disease: a unifying hypothesis. Gastroenterology.

[31] Farré R, Fornari F, Blondeau K (2010). Acid and weakly acidic solutions impair mucosal integrity of distal exposed and proximal non-exposed human oesophagus. Gut.

[32] Quigley EM (2006). Non-erosive reflux disease, functional heartburn and gastroesophageal reflux disease; insights into pathophysiology and clinical presentation. Chin J Dig Dis.

[33] Nagahama K, Matsunaga Y, Kawachi M (2012). Acotiamide, a new orally active acetylcholinesterase inhibitor, stimulates gastrointestinal motor activity in conscious dogs. Neurogastroenterol Motil.

